# Unravelling Tumour Microenvironment in Melanoma at Single-Cell Level and Challenges to Checkpoint Immunotherapy

**DOI:** 10.3390/genes13101757

**Published:** 2022-09-28

**Authors:** Xinyu Bai, Camelia Quek

**Affiliations:** Faculty of Medicine and Health, The University of Sydney, Sydney, NSW 2006, Australia

**Keywords:** melanoma, single-cell, transcriptomics, sequencing, spatial, immunotherapy, cancer, treatment, diagnosis

## Abstract

Melanoma is known as one of the most immunogenic tumours and is often characterised by high mutation burden, neoantigen load and immune infiltrate. The application of immunotherapies has led to impressive improvements in the clinical outcomes of advanced stage melanoma patients. The standard of care immunotherapies leverage the host immunological influence on tumour cells, which entail complex interactions among the tumour, stroma, and immune cells at the tumour microenvironmental level. However, not all cancer patients can achieve a long-term durable response to immunotherapy, and a significant proportion of patients develops resistance and still die from their disease. Owing to the multi-faceted problems of tumour and microenvironmental heterogeneity, identifying the key factors underlying tumour progression and immunotherapy resistance poses a great challenge. In this review, we outline the main challenges to current cancer immunotherapy research posed by tumour heterogeneity and microenvironment complexities including genomic and transcriptomic variability, selective outgrowth of tumour subpopulations, spatial and temporal tumour heterogeneity and the dynamic state of host immunity and microenvironment orchestration. We also highlight the opportunities to dissect tumour heterogeneity using single-cell sequencing and spatial platforms. Integrative analyses of large-scale datasets will enable in-depth exploration of biological questions, which facilitates the clinical application of translational research.

## 1. The Emergence of Single-Cell Sequencing and Spatial Platforms to Dissect Melanoma Microenvironment

In recent years, the emergence of single-cell sequencing and spatially resolved multi-omics platforms have rapidly advanced the field of immuno-oncology research. These technologies brought higher resolution to characterise the cellular composition and transcriptional programs within the tumour microenvironment (TME), offering new opportunities to uncover rare cell types, infer cellular interactions and identify molecular targets, hence offering better understandings of the mechanisms of immunotherapy response and resistance. In this section, we provide a brief overview of single-cell platforms that are widely used in cancer immunotherapy research.

### 1.1. Single-Cell Sequencing

Single-cell sequencing platforms are subdivided into cell-dissociated (individual cells are sorted prior to sequencing) and spatially resolved methods (map the location of the transcripts in an intact tissue section). Technologies that sequence the dissociated cells are broadly classified by their respective cell isolation methods. Droplet encapsulation methods, including 10x Genomics Chromium and Dolomite-Bio Nadia, are popular platforms for the sequencing of tumour dissociate samples due to the high number of sorted cells, allowing for high-throughput and relatively higher cost-effectiveness [1,2]. Microwell encapsulation platforms such as Fluidigm C1, Illumina/Bio-Rad ddSeq, Takara-Bio ICell8 and BD Rhapsody are commonly available platforms that have been developed over time. These platforms can vary in cost, labour, and sample size, and would thus be selective for different studies in cancer research. Since tumour samples often require the platform to accommodate for a range of cell size due to the multitude of cell types within the TME, more recent platforms such as the Rapsody and Illumina/Bio-Rad are more popular in this field [3]. Lastly, traditional fluorescence-activated cell sorting (FACS) based single-cell sequencing approaches such as SMART-seq2 [4] and MARS-seq [5] are well-established techniques in the laboratory. FACS is unrestricted by the size and morphology of the cells and total cell numbers, facilitating the selection of rare cell types from tumour samples. However, FACS entails staining the cell dissociate with pre-selected antibodies, and thus the identification of cell subtypes requires marker multiplexing for accurate cell sorting and downstream analysis [3].

### 1.2. Spatially Resolved Genomics and Transcriptomics

The TME is a heterogenous tissue architecture containing not only the tumour cells, but also a variety of non-immune and immune cells within the microenvironment. Each cell is constantly communicating with other cells and this in turn influences the function and fate of the cell. Furthermore, intracellular organisations of DNA and RNA can provide critical clues to the gene expression and post-transcriptional regulation underlying drug resistance. Thus, the spatial organisation of cells is critical for the accurate interpretation of tissue functions and cellular interactions. Single-cell spatial omics technologies have been developed to preserve the tissue context which retain the cell location information that helps to provide further insights into the interplay between tumour and the microenvironmental cells.

Two classes of methods have been popularised for imaging-based single-cell genomics and transcriptomics, based on multiplexed fluorescence in situ hybridisation (FISH) or in situ sequencing. FISH has been well-established for the visualisation and quantification of RNA at the single-molecule level [6,7], and multiplexed FISH, using combinatorial fluorescent colours, is developed for the gene-expression profiling of single-cells [8]. But the limited number of distinct colour channels posed a challenge to expanding the number of detectable targets for genome-scale imaging. Until this date, multiplexed error-robust (FISH) MERFISH has been used, which implements combinatorial labelling detected by sequential imaging and error robust binary coding schemes [9]. MERFISH has since been extended to DNA imaging, which enables simultaneous imaging of the 3D organisation of the chromatin at the genome scale [10]. Another multiplexed FISH method known as seqFISH+ uses sequential colour codes to detect RNAs and a binary code error-correction scheme akin to MERFISH. Both MERFISH and seqFISH+ have reported the imaging of transcripts for 10,000 genes in single cells [11,12]. Overall, multiplexed FISH approaches provide in situ single-cell transcriptomics and 3D genome analyses with high spatial resolution.

Imaging-based in situ sequencing approaches for single cell transcriptomic analysis are developed in parallel, and can be performed in either a targeted or untargeted manner for hypothesis-testing or empirical studies in cancer research. For targeted in situ sequencing, Ke et al. first described a method based on padlock probing, rolling-circle amplification and sequencing-by-ligation chemistry that can be applied to fixed cells and tissues [13]. More recent adaptations have used a multitude of methods to improve the multiplexity or detection efficiency of in situ sequencing, such as eliminating the inefficient RNA-to-cDNA conversion step, improving sequencing accuracy, using hydrogel-based clearing or sample expansion, combining with FISH, or using more efficient gap-filling enzymes [14,15,16,17]. An example is STARmap which integrates hydrogel-tissue chemistry, targeted signal amplification and sequencing method to achieve simultaneous imaging of up to 1020 targeted genes with a detection efficiency comparable to that of single-cell RNA sequencing [14]. In untargeted in situ sequencing, FISSEQ, can achieve genome-wide coverage and the detection of >8000 genes in the same sample [14].

Spatial transcriptomic analysis can also be achieved by spatially resolved RNA capture followed by sequencing. This was first demonstrated by Ståhl et al., where spatially barcoded and oligo(dT) probes are printed as microarrays of spots on the surface of glass slides [18]. After placing the tissue sections on the array, enzymic permeabilisation releases the mRNA from the tissue, which becomes free to hybridise with surface probes. Initial microarrays consisted of spots of ~100 μm in diameter, which captured a mix of tens of cells for transcriptomic profiling. This spatial transcriptomic technology was further developed and commercialised as 10x Genomics Visium, which increased the sensitivity and throughput by reducing the spot diameter to 55 μm. Depending on tissue type and thickness, the resolution was improved to offer an average gene expression profile of 1–10 cells in a single spot. To enable single cell resolution, Slide-seq [19] and HDST [20] are developed to capture cellular and biological expression at a single-cell level. Slide-seq operates on a monolayer of 10-μm beads, with each bead decoded by SOLiD [21] sequencing chemistry, to identify its spatial location. In HDST, the spatial resolution was improved to subcellular levels by packing 2-μm beads together, with a sequential hybridisation strategy to decode the spatial barcodes [20]. As sequencing technology advances, multimodal spatial profiling is becoming possible. The introduction of new in situ barcoding schemes for more biomolecules will yield more layers of molecular information from the same tissue. Combinations of barcoding strategies will enable spatial multi-omics approaches to retrieve information about splice variants, non-coding RNA, genetic and epigenetic alterations and proteins, all in a single experiment [22]. Some of these layers are already being added to the transcriptomic profiling of tissues, such as SM (Spatial Multi-omics)-Omics [23] and DBiT-seq [24], which offer parallel spatial transcriptomic profiling and antibody-based protein barcoding.

Integrated analyses of single-cell sequencing and spatial multi-omics are revealing TME details of cellular and subcellular organisation at an unprecedented level. Applications of these technologies have demonstrated exciting results, such as the generation of highly granular and functionally annotated spatial cell atlases [25], identification of potential ligand-receptor and paracrine signalling [26], and the provision of insights into tumour and immune cell evolution [27]. Future expansion of these technologies offers promising opportunities to study tumour heterogeneity. Novel signalling pathways unravelled by non-targeted sequencing can be screened for new therapeutic targets. Interaction events identified by ligand-receptor analyses will be stratified by pairs of spatially colocalised cells, thereby providing a more accurate view of routes of communication that will, for instance, facilitate the differentiation of effector and bystander cells in the TME. Trajectory analysis algorithms can also take advantage of the spatial positions of cells in associated transitional stages with cell-cell interaction events or spatial niches within the tissue environment.

## 2. Insights into Heterogeneity of Tumour Microenvironment from Single-Cell Expression Profiling

### 2.1. Inter- and Intra-Tumoural Heterogeneity

Tumour heterogeneity is a major driver of cancer progression, treatment resistance, and recurrences [28,29]. The heterogeneity of tumours and TME is broadly categorised into inter-tumour heterogeneity and intra-tumour heterogeneity [30,31,32].

Inter-tumoural heterogeneity (also known as inter-lesion heterogeneity) refers to the variations of different tumour genotypes among patients, even within the same histological subtypes [32,33]. Upon evaluating the single-cell transcriptional program from 19 metastatic melanoma tumours, Tirosh et al. identified a rare therapy-resistant subpopulation of melanoma cells expressing high levels of AXL Receptor Tyrosine Kinase inside the heterogenous tumours harbouring MITF (microphthalmia-associated transcription factor) due to clonal selection after targeted treatment with RAF/MEK inhibition [34]. MITF is an established contributor of melanoma heterogeneity through its multi-pathway influence on melanocyte differentiation, proliferation, and metastatic potential. In particular, low MITF in melanoma was shown to induce phenotype switching as an invasive mechanism [35], and thus the changing expression of this transcription factor can lead to the subsequent generation of resistant melanoma subpopulations [36,37]. In another cohort of patients with triple negative breast cancer, Wu et al. observed that the non-immune cell population, including cancer-associated fibroblasts in the complex stromal compartment, was functioning as an immune checkpoint for molecule expression, and its close interactions with T lymphocytes through lymphocyte activation and complement pathways were vital in regulating anti-tumour immunity [25].

Intra-tumoural heterogeneity (also known as intra-lesion heterogeneity) refers to the presence of distinct subpopulations of cancer cells with unique genetic traits in the single tumour of a patient, which can exhibit across different spatial locations of a tumour or evolve over time [32,38]. By inferring the genomic and transcriptional state changes of the TME and non-neoplastic cells among primary and metastatic tumours in patients with uveal melanoma (UM), Durante et al. found that class 1 UM subclones with low metastatic rate and signature driver mutations (EIF1AX and SF3B1) continue to evolve with the development of canonical/non-canonical aberrations that contribute to tumour progression [27]. Class 2 UM subclones with BAP1 inactivating mutations create an immunosuppressive microenvironment that facilitates metastasis through immune evasion [27]. Intra-tumoural heterogeneity was also observed in the transcriptomes of single cells from matched primary tumours and lymph node metastases in head and neck squamous cell carcinoma (HNSCC) patients. Puram et al. identified that tumour cells varied within patients in their expression of signatures strongly related to partial epithelial-to-mesenchymal transition (p-EMT), promoting resistance to anti-tumour immunity through dampening T lymphocyte-specific cytokines or regulating the expression of multiple inhibitory immune checkpoint molecules [39]. Such underlying heterogeneity provides deeper insights into one of the major causes of patients who either fail to respond to immunotherapy initially, or experience relapse after treatment.

### 2.2. The Spectrum of Spatial and Temporal Intra-Tumoural Heterogeneity

Heterogeneity in the tumour is not only limited to cancer cells, but also influences peritumoural stroma cells, tumour-infiltrating lymphocytes, and other stimulatory/inhibitory elements [33,40]. This heterogeneity includes spatial heterogeneity, which describes the non-uniform distribution of genetically diverse clonal populations across different disease sites or within a single site or tumour, and temporal heterogeneity, a term applied to polyclonal features of an individual tumour that evolve over time (i.e., heterogeneity between the primary tumour and a subsequent local or distant recurrence within a patient) [28,41,42].

Spatial heterogeneity is often defined as the differences in features such as genomics and cell morphology within an individual primary or metastatic tumour (intra-tumoural), and between different tumours within the same individual (intra-patient). In a retrospective cohort study, Jerby-Arnon et al. revealed a resistance programme associated with T cell exclusion and cyclin-dependent kinases (CDK4 and CDK6) using single-cell RNA sequencing from 33 melanoma patients, and further validated on 90 samples from 26 patients with multiple biopsies per patient [43]. The inter-patient differences in the therapy-resistant expression programs were larger than intra-patient, implying the intrinsic differences that reflect different treatment responses and outcomes [43]. In addition, a prospective cohort study of 327 tumour regions from 100 cases of early stage non-small-cell lung cancer highlighted that more than 75% of driver mutations in somatic cells were broadly heterogenous and were inconsistent in all samples when metastases were detected in patients with the same tumour [44]. The extent of spatial heterogeneity of molecular and cellular features within an individual tumour affects the utility of bulk gene expression signatures as markers associated with poor prognosis or therapy-resistant biomarkers.

Temporal heterogeneity refers to the initial (primary) tumour cells that have evolved with different mutational processes and selective pressures, resulting in variations of molecular composition of the tumours. The tumour cells also affect the intravascular cells, immune cells, stroma cells, and other cell types that constitute the complex TME [29,45]. The tumour immune compartment, along with the clonal selection of tumour cells, can influence response to anti-tumour treatments [29]. Analysis of single-cell transcriptomic profiles of 32 melanoma patients before and after checkpoint-based treatment indicated that the transitions between exhausted and memory effector T cell states were reflective of tumour regression, and the presence of CD8+ T cells with a specific transcription factor, TCF7, was identified as a predictive marker of response for immunotherapy [46]. Furthermore, the tracking of T cell clones and their transcriptional phenotypes from patients with basal or squamous cell carcinoma before and after anti-PD-1 therapy, highlighted the fact that the clonal expansion of neoantigen-specific T cells from the peripheral compartment were associated with responses to immunotherapy [47].

## 3. Clinical Considerations of Tumour Microenvironment Heterogeneity in Response and Resistance to Checkpoint-Based Immunotherapies

### 3.1. Impact of Tumour and Immune Microenvironment Heterogeneity on Response to Immunotherapies

In recent years, a plethora of evidence has demonstrated that the genetic and immunological heterogeneity in the TME influences the likelihood of malignant cells surviving immunotherapies in patients with solid tumours, including melanoma [3,33,40,43,46]. Achieving objective responses to immune checkpoint therapies can vary, based on the neoantigen and immunological profiles of the TME [48]. For instance, patients with high intra-tumoural heterogeneity of neoantigens that responded to T cell-antitumoural immunity had decreased fractions of clonal immunogenic neoantigens and generally responded less to T cells during immune checkpoint therapies [49]. Experimental mouse model studies also demonstrated that a homogenous population of immunogenic neoantigens was critical to the success of responsiveness to immunotherapies [38,50,51]. In the CA209-038 study of 68 patients with advanced melanoma who progressed on ipilimumab before and after nivolumab treatment, cytolytic activity was increased during treatment and represented the immune-inflamed environment (a high level of T cell infiltration) that enriched pre-treated tumours of anti-PD-1 responders [52]. However, in another cohort of the study, tumours with high cytolytic activity and interferon-γ scores failed to associate with clinical responses to anti-PD-1 therapy [40]. Jerby-Arnon et al. raised the difficulty of using bulk genomic and transcriptomic data to recover intracellular programmes of malignant cells, resulting in the discrepancy of linking malignant cell states to high (immune-inflamed tumour) or low (non-inflamed tumour) levels of tumour-infiltrating T cells [43]. The integrative single-cell strategy has enabled Jerby-Arnon et al. to define an immune-exclusion gene expression programs, including a cyclin-dependent kinase pathway to differentiate immune-inflamed from non-inflamed tumours [43]. Dissecting heterogeneity in the TME at single cell resolution will be necessary to fully understand tumour behaviour and its response to treatment.

### 3.2. Heterogenous Responses in Patients with Innate and Acquired Resistance

The patterns of treatment response heterogeneity are becoming more relevant from a clinical point of view, especially for patients who relapse after treatment and develop disease progression or recurrence [53,54]. Drugs that target a single biological pathway may induce the emergence of new mutation(s) in the tumour cells, favouring alternative pathways and further tumour progression [55,56]. These drugs can cause changes in the molecular composition of the tumour cells, leading to the dominance of therapy-resistant clones [57]. Non-responding patients with drug-resistant subpopulations of cells within the tumours can be categorised into two main groups: (i) primary or innate resistance refers to those who fail to respond to immunotherapies or have stable disease for less than 6 months before disease progression, and (ii) secondary or acquired resistance refers to those who relapse after an initial response to immunotherapies and develop disease progression [58,59,60]. Using a targeted single-cell approach, Kakavand et al. observed that the respective 28% and 22% of metastatic melanoma patients with acquired resistance to anti-PD-1 based therapies developed tumoural PTEN loss and impaired HLA-A regulation in the TME, implying tumours across patients achieved resistance in different ways [61]. Most of the current research on tumour heterogeneity of treatment responses has come from targeted drug therapies. In an early landmark study conducted by Tirosh et al. using Smart-seq2 protocol to dissect a heterogenous mixture of malignant cells expressing different levels of AXL kinase, it was identified that AXL-high cells were resistant in tumours treated with vemurafenib (BRAFi) or dabrafenib (BRAFi) and trametinib (RAF/MEKi), a mechanism that infers the use of checkpoint-based immunotherapies for patients who fail convectional targeted therapies [34]. Furthermore, since a low MITF/AXL ratio was recognised as a predictor of early resistance [36], microRNA (miRNA) regulation of MITF expression was also explored as a novel therapeutic target to reverse the resistant phenotype [62,63,64] among many other miRNA pathways dysregulated in melanoma [65]. MITF expression regulates melanogenesis via the active transcription of pigmentation genes, including *DCT*, which encodes for dopachrome tautomerase [66]. Ho et al. identified the upregulation of DCT in rare drug-resistant subpopulations of human melanoma cells, prior to BRAFi treatment, which was only present in the single-cell RNA analysis when compared to bulk RNA sequencing [67]. Another mechanism of resistance to targeted therapies in melanoma involves the intrinsic control of apoptotic pathway mediated by the overexpression of pro-survival members of the BCL-2 family, shown in cell culture studies [68,69]. Selective inhibition of these BCL-2 family proteins is being explored in potential combinatory targeted therapy drugs, and is further reviewed elsewhere [70]. Overall, the genetic and non-genetic features of heterogeneity present at baseline and acquired post-treatment must be considered in clinical decision making, and similar single-cell investigations should be performed on longitudinal patient samples undergoing immunotherapy to reveal new mechanisms of resistance.

## 4. Heterogenous Expression of PD-L1 in the Tumour-Immune Microenvironment

PD-L1 expression level is identified in either tumour or immune cells and is often a selection criterion for predicting clinical response to PD-1 or PD-L1 inhibitors across various solid tumour types, including melanoma [71,72]. Although PD-L1 diagnostic testing is now established as a routine clinical practice, the expression level of PD-L1 is heterogenous both within and among tumour sites on various spatial and temporal scales [73,74]. A plethora of studies, including the CheckMate 067 trial, highlight the fact that the level of PD-L1 expression alone is a poor predictive biomarker, for which approximately 20% of patients whose tumours have minimal or no PD-L1 expression may achieve an objective response to anti-PD-1/PD-L1 treatment [71,73,74,75,76]. Upon evaluating pre-treatment biopsies from melanoma patients treated with anti-PD-1 therapy in a phase I clinical trial, Shin et al. observed that the upregulation of surface PD-L1 expression through the interferon-γ receptor pathway was heterogenous in subclones harbouring defective Janus kinases JAK1 and JAK2 [77]. In addition, the discrepancy in PD-L1 expression was also identified in subclones within the tumour immune microenvironment harbouring impaired antigen processing and presentation machinery in patients receiving immunotherapies, and has been associated with poor clinical outcomes in melanoma and other cancer types such as lung cancer and colorectal carcinoma [78,79,80]. It is likely that the emerging heterogeneity of PD-L1 expression may explain why a subset of patients with tumours expressing PD-L1+ fail to respond, while some with PD-L1– negative neoplasms respond well to checkpoint-based immunotherapies.

## 5. Integrating Tumour Microenvironment Heterogeneity into Clinical Decision Making

The rapid advancements in single-cell sequencing and spatial technologies have shed light on modern cancer research, while the improved understanding of patient and tumour microenvironmental heterogeneity has brought new challenges to optimising therapeutic strategies. The field is moving towards translational applications of biological discoveries and has great potential in guiding clinical decision making.

As reviewed in the earlier sections, inter- and intra-tumoural heterogeneity encompasses a multitude of factors that define the unique biology of a tumour or tumour clone. These unique factors are driven by a combination of intrinsic and extrinsic changes which can be detected as variability at a multi-omics level. These changes can be leveraged using next-generation sequencing to reveal therapeutic vulnerabilities of cancer patients. This was demonstrated by the development of highly selective BRAF inhibitors (vemurafenib and dabrafenib) for melanoma patients after the identification of the target mutation [81,82]. Although the development of targeted therapies has transformed the treatment landscape for advanced melanoma, acquired resistance still poses a great challenge to improving patient outcome [59]. Over the years, several published works have identified the activation of compensatory pathways, which has led to the development of combinatory therapies such as dual BRAF/MEK inhibition [83,84]. However, the heterogeneity of resistance mechanisms presents a challenge in creating a rationally designed combination strategy that will be applicable for all patients. Previous studies have identified many more overlapping pathways with defined mechanisms of resistance, including NRAS, PI3K, PDGFR, IGF-1R, and GFR, among others [85,86]. A study analysing 132 melanoma samples that progressed with BRAF inhibitor treatment reported that a defined resistance mechanism was only identified in 58% of samples [85]. Further work using non-targeted sequencing methods is required to decipher novel mechanisms of therapy resistance and guide the development of therapeutic strategies.

In relation to discovering the features of TME heterogeneity and mechanisms of resistance, combinatory and personalised checkpoint immunotherapy strategies for melanoma are being actively researched. The CheckMate 067 trial demonstrated the superior efficacy of combination nivolumab plus ipilimumab over nivolumab or ipilimumab alone, though with increases in the rates of immune-related adverse events [87]. Clinical trials for other cancers have also validated the combination of immunotherapy and other therapeutic agents with independent anti-tumour effects, showing prolonged overall and progression-free survival in patients treated with combinatory regimens [88,89]. To overcome adaptive immunotherapy resistance in the TME, targeting other checkpoints including lymphocyte activation gene-3 (LAG-3), T cell immunoglobulin and ITIM domain (TIGIT), T cell immunoglobulin and mucin-domain containing-3 (TIM-3), V-domain immunoglobulin suppressor of T cell activation (VISTA), B7 homolog 3 protein (B7-H3), inducible T cell costimulator (ICOS), and B and T lymphocyte attenuator (BTLA) are currently being tested in multiple clinical trials [90], with those involving solid tumours summarised in Table 1. With the expansion of translational research and the validation of novel drug targets in clinical trials, alternative checkpoint inhibitors are becoming feasible and promising options for personalising immunotherapy for solid tumours.

The complex and dynamic signalling network within the TME can also provide insight into the degree of interactive heterogeneity seen in tumours. Inhibition of the RAS/RAF/MAPK pathway not only results in the upregulation of compensatory receptor tyrosine kinase pathways [91], but has also been shown to modulate the host immune response, including alterations in T-cell responses and the expression of PD-L1 [92,93]. A separate study reported that the loss of tumour suppressor PTEN was associated with T cell exclusion and an immunosuppressed TME [94]. These findings identified genetic changes with cellular and phenotypic features that facilitated crosstalk between the intrinsic and extrinsic factors in the modulation of TME heterogeneity. This also sparked interest in combining targeted agents and immunotherapy to offer both short- and long-term benefits to cancer patients, which led to multiple clinical trials where molecular testing strategies are employed for both patient stratification and biomarker discovery purposes [95,96].

The genotypic and phenotypic variability posed by inter- and intra-tumoral heterogeneity has shown promising potential in biological research, but several challenges remain in its integration into personalised drug design and clinical decision making. Firstly, a single tumour biopsy is a static and partial representation of the overall heterogeneity in a patient. Temporal analyses of multiple biopsies throughout the course of disease and treatment are required for the accurate depiction of spatial and temporal heterogeneity. Till date, repeated biopsies during and after treatment for disease progression have been incorporated into clinical trials [97,98]. Furthermore, the detection of tumour mutations in circulating tumour DNA has demonstrated feasibility and predictive values, which may be adapted as early diagnostic or relapse monitoring tools [99]. More research studies using non-targeted single-cell sequencing methods are also required to expand the understanding of rare patterns of heterogeneity and therapy resistance, and to guide novel drug development. Large scale collaborative efforts such as the Human Tumour Atlas Network [100] and the Human BioMolecular Atlas Program [101] are working towards combined databases of highly-multiplexed tissue imaging and single-cell omics data, to offer comprehensive cellular information in the context of cancer. The integration of spatial, single-cell and phenotypic datasets will empower a deeper understanding of heterogeneity at genomic, transcriptomic, proteomic and spatial organisation levels.

## 6. Conclusions

Heterogeneity of the tumour and microenvironment complexity are the main contributors to different treatment responses and clinical outcomes. The single-cell approach has contributed to the study of tumour heterogeneity and drug resistance arising from the TME. The advancement in single cell and spatial multi-omics platforms, as well as the integrated analysis of these large-scale datasets, are offering unprecedented powers to identify the intrinsic, extrinsic and interactive processes driving the development of tumour heterogeneity. The identification of the key and rare subpopulations of cells and molecular processes will aid in improving diagnosis and novel drug development for cancer patients.

## Figures and Tables

**Table 1:** Current clinical trials of novel and combinatory checkpoint inhibitor drugs for solid tumours.

Target	Drug	Clinical Trial ID	Phase	Settings	Tumour Types	Treatment Arms	Status
LAG-3	Eftilagimod α (IMP321)	NCT03252938	1	Advanced/metastatic	Solid tumours	Eftilagimod α	Recruiting
		NCT00324623	1	Advanced/metastatic	Melanoma	Cyclophosphamide, fludarabine followed by melan-A VLP vaccine and eftilagimod α	Completed
		NCT00365937	1	Adjuvant	Melanoma	Eftilagimod α ± HLA-A2 peptides	Terminated
		NCT01308294	1, 2	Stage II-1V	Melanoma	Eftilagimod α + tumour antigenic peptides + monatide	Terminated
	Relatlimab (BMS-986016)	NCT02966548	1	Advanced/metastatic	Solid tumours	Relatlimab ± nivolumab	Active, not recruiting
		NCT01968109	1	First. second line	Solid tumours	Relatlimab ± nivolumab	Active, not recruiting
		NCT03743766	2	Advanced/metastatic	Melanoma	Relatlimab + nivolumab	Recruiting
		NCT03470922	2, 3	Advanced/metastatiC	Melanoma	Relatlirnab ± nivolumab	Active, not recruiting
		NCT03335540	1, 2	Advanced/metastatic	Sold tumours	Relatlimab + nivolumab or cabiralizumab or ipilimumab or IDO1 inhibitor or radiation therapy	Active, not recruiting
		NCT02519322	2	Neoadjuvant and adjuvant	Melanoma	Nivolumab ± relatlimab or ipilimumab	Recruiting
		NCT03459222	2	Advanced/metastatic	Solid tumours	Relatlimab + nivolumab + IDO1 inhibitor or relatlimab + nivolumab + ipilimumab	Recruiting
	LAG525	NCT02460224	1, 2	Advanced/metastatic	Solid tumours	LAG525 ± spartalizumab (anti-PD-1)	Completed
		NCT03365791	2	Advanced/metastatic	Solid or hematologic malignancy	LAG525 + spartalizumab (anti-PD-1)	Completed
	Fianlimab (REGN3767)	NCT03005782	1	Advanced/metastatic	Solid tumours or lymphomas	Fianlimab ± cemiplimab (anti-PD-1)	Active, not recruiting
	BI 754111	NCT03433898	1	Advanced/metastatic	Solid tumours	BI 754111 ± BI 754091 (anti-PD-1)	Active, not recruiting
		NCT03156114	1	Advanced/metastatic	Solid tumours	BI 754111 ± BI 754091 (anti-PD-1)	Active, not recruiting
		NCT03697304	2	Advanced/metastatic	Solid tumours	BI 754111 or BI 836880 (bispecific VEGF and Ang2 Ab) + BI 754091 (anti-PD-1)	Active, not recruiting
		NCT03964233	1	Advanced/metastatic	Solid tumours	BI 754111 + BI 754091 ± BI 907828 (MDM2-p53 antagonist)	Recruiting
	Sym022	NCT03489369	1	Advanced/metastatic	Solid tumours or lymphomas	Sym022	Completed
		NCT03311412	1	Advanced/metastatic	Solid tumours or lymphomas	Sym022 + Sym021 (anti-PD-1) ± Sym023 (anti-TIM-3)	Completed
	MGD013	NCT03219268	1	Advanced/metastatic	Solid or hematologic malignancy	MGD013 + margetuximab (anti-HER2 monoclonal antibody)	Active, not recruiting
	TSR-033	NCT03250832	1	Advanced/metastatic	Solid tumours	TSR-033 ± dostarlimab ± mFOLFOX6 or FOLFIRI ± bevacizumab	Active, not recruiting
	INCAGN02385	NCT03538028	1	Advanced/metastatic	Solid tumours	INCAGN02385	Completed
		NCT04370704	1, 2	Advanced/metastatic	Solid tumours	INCAGN02385 + INCAGN02390 (Anti-TIM-3) ± INCMGA00012 (anti-PD-1)	Recruiting
	XmAb22841	NCT03849469	1	Advanced/metastatic	Solid tumours	XmAb22841 ± pembrolizumab	Active, not recruiting
	LBL-007	NCT04640545	1	Advanced/metastatic	Melanoma	LBL-007 + toriparimab (anti-PD-1)	Recruiting
	FS118	NCT03440437	1	Advanced/metastatic	Solid or hematologic malignancy	FS118	Recruiting
	RO7247669	NCT04140500	1	Advanced/metastatic	Solid tumours	RO7247669	Recruiting
	EMB-02	NCT04618393	1, 2	Advanced/metastatic	Solid tumours	EMB-02	Recruiting
TIGIT	Tiragolumab (MTIG7192A/RG-6058)	NCT02794571	1	Locally advanced or metastatic	Solid tumours	Tiragolumab ± atezolizumab ± chemotherapy	Recruiting
	Vibostolimab (MK-7684)	NCT02964013	1	Advanced/metastatic	Solid tumours	Vibostolimab ± pembrolizumab ± pemetrexed/carboplatin: carboplatin + cisplatin + etoposide	Active, not recruiting
		NCT04305054	1, 2	First line	Melanoma	Pembrolizumab ± vibostolimab or quavonlimab (MK-1308) ± lenvatinib	Recruiting
		NCT04305041	1, 2	Stage IV	Melanoma	Pembrolizumab ± guavonlimab + vibostolimab or lenvatinib	Recruiting
		NCT04303169	1, 2	Stage III	Melanoma	Pembrolizumab ± vibostolimab or V937 (oncolytic virus)	Recruiting
	OMP-313M32	NCT03119428	1	Locally advanced or metastatic	Solid tumours	OMP-313M32 ± nivolumab	Terminated
	BMS-986207	NCT02913313	1, 2	Advanced/metastatic	Solid tumours	BMS-986207 ± nivolumab ± ipilimumab	Recruiting
		NCT04570839	1, 2	Advanced/metastatic	Solid tumours	BMS-986207 + nivolumab + COM701 (anti-PVRIG Ab)	Recruiting
	Domvanalimab (AB-154)	NCT03628677	1	Advanced/metastatic	Solid tumours	Dombvanalimab + zimberelimab (AB122, anti-PD-1)	Active, not recruiting
	ASP8374	NCT03945253	1	Advanced/metastatic	Solid tumours	ASP8374	Completed
		NCT03260322	1	Advanced/metastatic	Solid tumours	ASP8374 ± pembrolizumab	Completed
	IBI939	NCT04353830	1	Advanced/metastatic	Solid tumours	IBI939 ± sintilimab (anti-PD-1)	Recruiting
	Ociperlimab (BGB-A1217)	NCT04047862	1	Advanced/metastatic	Solid tumours	Ociperlimab + tislelizumab (anti-PD-1) ± chemotherapy	Recruiting
	COM902	NCT04354246	1	Advanced/metastatic	Solid tumours	COM902 ± COM701 (anti-PVRIG Ab)	Recruiting
	M6223	NCT04457778	1	Advanced/metastatic	Solid tumours	M6223 ± bintrafusp alfa (M7824, bispecific TGF-β and PD-L1 Ab)	Recruiting
TIM-3	Sym023	NCT03489343	1	Advanced/metastatic	Solid tumours or lymphomas	Sym023	Completed
	LY3321367	NCT03099109	1	Advanced/metastatic	Solid tumours	LY3300054 (anti-PD-L1) + LY3321367	Active, not recruiting
		NCT02791334	1	Advanced/metastatic	Solid tumours	LY3300054 (anti-PD-L1) ± LY3321367 or abemaciclib or ramucirumab or merestinib	Active, not recruiting
	Cobolimab (TSR-022)	NCT02817633	1	Advanced/metastatic	Solid tumours	Cobolimab ± nivolumab or TSR-042 (anti-PD-1) ± TSR-033 or chemotherapy	Recruiting
		NCT03307785	1	Advanced/metastatic	Solid tumours	Dostarlimab (TSR-042, anti-PD-1) ± TSR-022 + chemotherapy	Active, not recruiting
		NCT04139902	2	Ncoadjuvant	Melanoma	Cobolimab ± dostarlimab (TSR-042, anti-PD-1)	Recruiting
	Sabatolimab (MBG453)	NCT02608268	1, 2	Advanced/metastatic	Solid tumours	Sabatolimab ± PDR001 vs. chemotherapy	Active, not recruiting
	INCAGN02390	NCT03652077	1	Advanced/metastatic	Solid tumours	INCAGN02390	Completed
	BMS-986258	NCT03446040	1, 2	Advanced/metastatic	Solid tumours	BMS-986258 + nivolumab or rHuPH20	Active, not recruiting
	SHR-1702	NCT03871855	1	Advanced/metastatic	Solid tumours	SHR-1702 ± camrelizumab (anti-PD-1)	Unknown
	RO7121661	NCT03708328	1	Advanced/metastatic	Solid tumours	RO7121661	Active, not recruiting
B7-H3	Enoblituzumab (MGA271)	NCT01391143	1	Advanced/metastatic	Solid tumours	Enoblituzumab (MGA271)	Completed
		NCT02381314	1	Advanced/metastatic	Solid tumours	Enoblituzumab + ipilimumab	Completed
		NCT02475213	1	Advanced/metastatic	Solid tumours	Enoblituzumab + pembrolizumab or retifanlimab (MGA012, anti-PD-1)	Completed
	DS-7300a	NCT04145622	1, 2	Advanced/metastatic	Solid tumours	DS-7300a	Recruiting
	Orlotamab (MGD009)	NCT02628535	1	Advanced/metastatic	solid tumours	Orlotamab (MGD009)	Terminated
		NCT03406949	1	Advanced/metastatic	Solid tumours	Orlotamab + retifanlimab (MGA012, anti-PD-1)	Completed
	4SCAR-276	NCT04432649	1	Advanced/metastatic	Solid tumours	4SCAR-276	Recruiting
VISTA	JNJ-61610588	NCT02671955	1	Advanced/metastatic	Solid tumours	JNJ-61610588	Terminated
	CI-8993	NCT04475523	1	Advanced/metastatic	Solid tumours	CI-8993	Recruiting
	CA-170	NCT02812875	1	Advanced/metastatic	Solid tumours or lymphomas	CA-170	Completed
ICOS	Feladilimab	NCT03693612	2	Advanced/metastatic	Solid tumours	Feladilimab + tremelimumab (anti-CTLA-4) vs. chemotherapy	Completed
	JTX-2011	NCT02904226	1, 2	Advanced/metastatic	Solid tumours	JTX-2011 ± pembrolizumab or nivolumab or ipilimumab	Completed
	KY1044	NCT03829501	1, 2	Advanced/metastatic	Solid tumours	KY1044 ± atezolizumab	Recruiting
BTLA	INBRX-106	NCT04198766	1	Locally advanced or metastatic	Solid tumours	INBRX-106 ± pembrolizumab	Recruiting
	Cudarolimab (IBI101)	NCT03758001	1	Advanced/metastatic	Solid tumours	Cudarcenab ± sintilimab (anti-PD-1)	Active, not recruiting
	PF-04518600	NCT02315066	1	Advanced/metastatic	Solid tumours	PF-04518600 ± utomilumab (PF-05082566, agonist anti-TNFRSF9 Ab)	Completed
	TAB004 (JS004)	NCT04137900	1	Advanced/metastatic	Solid tumours or lymphomas	TAB004 ± toripalimab (anti-PD-1)	Recruiting
		NCT04278859	1	Advanced/metastatic	Solid tumours	TAB004 (JS004)	Unknown

## Data Availability

Not applicable.

## References

[1] Nguyen A., Khoo W.H., Moran I., Croucher P., Phan T.G. (2018). Single Cell RNA Sequencing of Rare Immune Cell Populations. Front. Immunol..

[2] Valihrach L., Androvic P., Kubista M. (2018). Platforms for Single-Cell Collection and Analysis. Int. J. Mol. Sci..

[3] Quek C., Bai X., Long G.V., Scolyer R.A., Wilmott J.S. (2021). High-Dimensional Single-Cell Transcriptomics in Melanoma and Cancer Immunotherapy. Genes.

[4] Picelli S., Bjorklund A.K., Faridani O.R., Sagasser S., Winberg G., Sandberg R. (2013). Smart-seq2 for sensitive full-length transcriptome profiling in single cells. Nat. Methods.

[5] Jaitin D.A., Kenigsberg E., Keren-Shaul H., Elefant N., Paul F., Zaretsky I., Mildner A., Cohen N., Jung S., Tanay A. (2014). Massively Parallel Single-Cell RNA-Seq for Marker-Free Decomposition of Tissues into Cell Types. Science.

[6] Femino A.M., Fay F.S., Fogarty K., Singer R.H. (1998). Visualization of single RNA transcripts in situ. Science.

[7] Raj A., van den Bogaard P., A Rifkin S.A., van Oudenaarden A., Tyagi S. (2008). Imaging individual mRNA molecules using multiple singly labeled probes. Nat. Methods.

[8] Levsky J.M., Shenoy S.M., Pezo R.C., Singer R.H. (2002). Single-cell gene expression profiling. Science.

[9] Chen K.H., Boettiger A.N., Moffitt J.R., Wang S., Zhuang X. (2015). RNA imaging. Spatially resolved, highly multiplexed RNA profiling in single cells. Science.

[10] Su J.H., Zheng P., Kinrot S.S., Bintu B., Zhuang X. (2020). Genome-Scale Imaging of the 3D Organization and Transcriptional Activity of Chromatin. Cell.

[11] Xia C., Fan J., Emanuel G., Hao J., Zhuang X. (2019). Spatial transcriptome profiling by MERFISH reveals subcellular RNA compartmentalization and cell cycle-dependent gene expression. Proc. Natl. Acad. Sci. USA.

[12] Eng C.-H.L., Lawson M., Zhu Q., Dries R., Koulena N., Takei Y., Yun J., Cronin C., Karp C., Yuan G.-C. (2019). Transcriptome-scale super-resolved imaging in tissues by RNA seqFISH+. Nature.

[13] Ke R., Mignardi M., Pacureanu A., Svedlund J., Botling J., Wählby C., Nilsson M. (2013). In situ sequencing for RNA analysis in preserved tissue and cells. Nat. Methods.

[14] Wang X., Allen W.E., Wright M.A., Sylwestrak E.L., Samusik N., Vesuna S., Evans K., Liu C., Ramakrishnan C., Liu J. (2018). Three-dimensional intact-tissue sequencing of single-cell transcriptional states. Science.

[15] Qian X., Harris K.D., Hauling T., Nicoloutsopoulos D., Manchado A.M., Skene N., Hjerling-Leffler J., Nilsson M. (2020). Probabilistic cell typing enables fine mapping of closely related cell types in situ. Nat. Methods.

[16] Liu S., Punthambaker S., Iyer E.P.R., Ferrante T., Goodwin D., Fürth D., Pawlowski A.C., Jindal K., Tam J.M., Mifflin L. (2021). Barcoded oligonucleotides ligated on RNA amplified for multiplexed and parallel in situ analyses. Nucleic Acids Res..

[17] Alon S., Goodwin D.R., Sinha A., Wassie A.T., Chen F., Daugharthy E.R., Bando Y., Kajita A., Xue A.G., Marrett K. (2021). Expansion sequencing: Spatially precise in situ transcriptomics in intact biological systems. Science.

[18] Ståhl P.L., Salmén F., Vickovic S., Lundmark A., Navarro J.F., Magnusson J., Giacomello S., Asp M., Westholm J.O., Huss M. (2016). Visualization and analysis of gene expression in tissue sections by spatial transcriptomics. Science.

[19] Rodriques S.G., Stickels R.R., Goeva A., Martin C.A., Murray E., Vanderburg C.R., Welch J., Chen L.M., Chen F., Macosko E.Z. (2019). Slide-seq: A scalable technology for measuring genome-wide expression at high spatial resolution. Science.

[20] Vickovic S., Eraslan G., Salmén F., Klughammer J., Stenbeck L., Schapiro D., Äijö T., Bonneau R., Bergenstråhle L., Navarro J.F. (2019). High-definition spatial transcriptomics for in situ tissue profiling. Nat. Methods.

[21] Gunderson K.L., Kruglyak S., Graige M.S., Garcia F., Kermani B.G., Zhao C., Che D., Dickinson T., Wickham E., Bierle J. (2004). Decoding randomly ordered DNA arrays. Genome Res..

[22] Larsson L., Frisén J., Lundeberg J. (2021). Spatially resolved transcriptomics adds a new dimension to genomics. Nat. Methods.

[23] Vickovic S., Lötstedt B., Klughammer J., Mages S., Segerstolpe Å., Rozenblatt-Rosen O., Regev A. (2022). SM-Omics is an automated platform for high-throughput spatial multi-omics. Nat. Commun..

[24] Liu Y., Yang M., Deng Y., Su G., Enninful A., Guo C.C., Tebaldi T., Zhang D., Kim D., Bai Z. (2020). High-Spatial-Resolution Multi-Omics Sequencing via Deterministic Barcoding in Tissue. Cell.

[25] Wu S.Z., Al-Eryani G., Roden D.L., Junankar S., Harvey K., Andersson A., Thennavan A., Wang C., Torpy J.R., Bartonicek N. (2021). A single-cell and spatially resolved atlas of human breast cancers. Nat. Genet..

[26] Zhuang X. (2021). Spatially resolved single-cell genomics and transcriptomics by imaging. Nat. Methods.

[27] Durante M.A., Rodriguez D.A., Kurtenbach S., Kuznetsov J.N., Sanchez M.I., Decatur C.L., Snyder H., Feun L.G., Livingstone A.S., Harbour J.W. (2020). Single-cell analysis reveals new evolutionary complexity in uveal melanoma. Nat. Commun..

[28] Nowell P.C. (1976). The clonal evolution of tumor cell populations. Science.

[29] Junttila M.R., de Sauvage F.J. (2013). Influence of tumour micro-environment heterogeneity on therapeutic response. Nature.

[30] McGranahan N., Favero F., de Bruin E.C., Birkbak N.J., Szallasi Z., Swanton C. (2015). Clonal status of actionable driver events and the timing of mutational processes in cancer evolution. Sci. Transl. Med..

[31] McGranahan N., Swanton C. (2017). Clonal Heterogeneity and Tumor Evolution: Past, Present, and the Future. Cell.

[32] Grzywa T.M., Paskal W., Wlodarski P.K. (2017). Intratumor and Intertumor Heterogeneity in Melanoma. Transl. Oncol..

[33] Jia Q.Z., Wang A., Yuan Y., Zhu B., Long H. (2022). Heterogeneity of the tumor immune microenvironment and its clinical relevance. Exp. Hematol. Oncol..

[34] Tirosh I., Izar B., Prakadan S.M., Wadsworth M.H., Treacy D., Trombetta J.J., Rotem A., Rodman C., Lian C., Murphy G. (2016). Dissecting the multicellular ecosystem of metastatic melanoma by single-cell RNA-seq. Science.

[35] Noguchi K., Dalton A.C., Howley B.V., McCall B., Yoshida A., Diehl J.A., Howe P.H. (2017). Interleukin-like EMT inducer regulates partial phenotype switching in MITF-low melanoma cell lines. PLoS ONE.

[36] Müller J., Krijgsman O., Tsoi J., Robert L., Hugo W., Song C., Kong X., Possik P.A., Cornelissen-Steijger P.D., Foppen M.H.G. (2014). Low MITF/AXL ratio predicts early resistance to multiple targeted drugs in melanoma. Nat. Commun..

[37] Simmons J.L., Pierce C.J., Al-Ejeh F., Boyle G.M. (2017). MITF and BRN2 contribute to metastatic growth after dissemination of melanoma. Sci. Rep..

[38] Wolf Y., Bartok O., Patkar S., Eli G.B., Cohen S., Litchfield K., Levy R., Jiménez-Sánchez A., Trabish S., Lee J.S. (2019). UVB-Induced Tumor Heterogeneity Diminishes Immune Response in Melanoma. Cell.

[39] Puram S.V., Tirosh I., Parikh A.S., Patel A.P., Yizhak K., Gillespie S., Rodman C., Luo C.L., Mroz E.A., Emerick K.S. (2017). Single-Cell Transcriptomic Analysis of Primary and Metastatic Tumor Ecosystems in Head and Neck Cancer. Cell.

[40] Hugo W., Zaretsky J.M., Sun L., Song C., Moreno B.H., Hu-Lieskovan S., Berent-Maoz B., Pang J., Chmielowski B., Cherry G. (2017). Genomic and Transcriptomic Features of Response to Anti-PD-1 Therapy in Metastatic Melanoma. Cell.

[41] Kleppe M., Levine R.L. (2014). Tumor heterogeneity confounds and illuminates: Assessing the implications. Nat. Med..

[42] Rodriguez-Meira A., Buck G., Clark S.-A., Povinelli B.J., Alcolea V., Louka E., McGowan S., Hamblin A., Sousos N., Barkas N. (2019). Unravelling Intratumoral Heterogeneity through High-Sensitivity Single-Cell Mutational Analysis and Parallel RNA Sequencing. Mol. Cell.

[43] Jerby-Arnon L., Shah P., Cuoco M.S., Rodman C., Su M.-J., Melms J.C., Leeson R., Kanodia A., Mei S., Lin J.-R. (2018). A Cancer Cell Program Promotes T Cell Exclusion and Resistance to Checkpoint Blockade. Cell.

[44] Jamal-Hanjani M., Wilson G.A., McGranahan N., Birkbak N.J., Watkins T.B.K., Veeriah S., Shafi S., Johnson D.H., Mitter R., Rosenthal R. (2017). Tracking the Evolution of Non-Small-Cell Lung Cancer. N. Engl. J. Med..

[45] Ge R., Wang Z., Cheng L. (2022). Tumor microenvironment heterogeneity an important mediator of prostate cancer progression and therapeutic resistance. NPJ Precis. Oncol..

[46] Sade-Feldman M., Yizhak K., Bjorgaard S.L., Ray J.P., de Boer C.G., Jenkins R.W., Lieb D.J., Chen J.H., Frederick D.T., Barzily-Rokni M. (2018). Defining T Cell States Associated with Response to Checkpoint Immunotherapy in Melanoma. Cell.

[47] Yost K.E., Satpathy A.T., Wells D.K., Qi Y., Wang C., Kageyama R., McNamara K.L., Granja J.M., Sarin K.Y., Brown R.A. (2019). Clonal replacement of tumor-specific T cells following PD-1 blockade. Nat. Med..

[48] Fridman W.H., Pages F., Sautes-Fridman C., Galon J. (2012). The immune contexture in human tumours: Impact on clinical outcome. Nat. Rev. Cancer.

[49] Fang W., Jin H., Zhou H., Hong S., Ma Y., Zhang Y., Su X., Chen L., Yang Y., Xu S. (2021). Intratumoral heterogeneity as a predictive biomarker in anti-PD-(L)1 therapies for non-small cell lung cancer. Mol. Cancer.

[50] Gejman R.S., Chang A.Y., Jones H.F., DiKun K., Hakimi A.A., Schietinger A., Scheinberg D.A. (2018). Rejection of immunogenic tumor clones is limited by clonal fraction. eLife.

[51] Milo I., Bedora-Faure M., Garcia Z., Thibaut R., Périé L., Shakhar G., Deriano L., Bousso P. (2018). The immune system profoundly restricts intratumor genetic heterogeneity. Sci. Immunol..

[52] Riaz N., Havel J.J., Makarov V., Desrichard A., Urba W.J., Sims J.S., Hodi F.S., Martín-Algarra S., Mandal R., Sharfman W.H. (2017). Tumor and Microenvironment Evolution during Immunotherapy with Nivolumab. Cell.

[53] Fusi A., Berdel R., Havemann S., Nonnenmacher A., Keilholz U. (2011). Enhanced detection of BRAF-mutants by pre-PCR cleavage of wild-type sequences revealed circulating melanoma cells heterogeneity. Eur. J. Cancer.

[54] Bradish J.R., Richey J.D., Post K.M., Meehan K., Sen J.D., Malek A.J., Katona T.M., Warren S., Logan T.F., Fecher L.A. (2015). Discordancy in BRAF mutations among primary and metastatic melanoma lesions: Clinical implications for targeted therapy. Mod. Pathol..

[55] Gide T.N., Quek C., Menzies A.M., Tasker A.T., Shang P., Holst J., Madore J., Lim S.Y., Velickovic R., Wongchenko M. (2019). Distinct Immune Cell Populations Define Response to Anti-PD-1 Monotherapy and Anti-PD-1/Anti-CTLA-4 Combined Therapy. Cancer Cell.

[56] Su Y., Wei W., Robert L., Xue M., Tsoi J., Garcia-Diaz A., Moreno B.H., Kim J., Ng R.H., Lee J.W. (2017). Single-cell analysis resolves the cell state transition and signaling dynamics associated with melanoma drug-induced resistance. Proc. Natl. Acad. Sci. USA.

[57] Gremel G., Lee R.J., Girotti M.R., Mandal A.K., Valpione S., Garner G., Ayub M., Wood S., Rothwell D., Fusi A. (2016). Distinct subclonal tumour responses to therapy revealed by circulating cell-free DNA. Ann. Oncol..

[58] Gide T.N., Pires da Silva I., Quek C., Ferguson P.M., Batten M., Shang P., Ahmed T., Menzies A.M., Carlino M.S., Saw R.P.M. (2021). Clinical and Molecular Heterogeneity in Patients with Innate Resistance to Anti-PD-1 +/− Anti-CTLA-4 Immunotherapy in Metastatic Melanoma Reveals Distinct Therapeutic Targets. Cancers.

[59] Gide T.N., Wilmott J.S., Scolyer R.A., Long G.V. (2018). Primary and Acquired Resistance to Immune Checkpoint Inhibitors in Metastatic Melanoma. Clin. Cancer Res..

[60] Sharma P., Hu-Lieskovan S., Wargo J.A., Ribas A. (2017). Primary, Adaptive, and Acquired Resistance to Cancer Immunotherapy. Cell.

[61] Kakavand H., Wilmott J.S., Menzies A.M., Vilain R., Haydu L.E., Yearley J.H., Thompson J.F., Kefford R.F., Hersey P., Long G.V. (2015). PD-L1 Expression and Tumor-Infiltrating Lymphocytes Define Different Subsets of MAPK Inhibitor-Treated Melanoma Patients. Clin. Cancer Res..

[62] Goswami S., Tarapore R.S., Poenitzsch Strong A.M., TeSlaa J.J., Grinblat Y., Setaluri V., Spiegelman V.S. (2015). MicroRNA-340-mediated degradation of microphthalmia-associated transcription factor (MITF) mRNA is inhibited by coding region determinant-binding protein (CRD-BP). J. Biol. Chem..

[63] Qian H., Yang C., Yang Y. (2017). MicroRNA-26a inhibits the growth and invasiveness of malignant melanoma and directly targets on MITF gene. Cell Death Discov..

[64] Haflidadóttir B.S., Bergsteinsdóttir K., Praetorius C., Steingrímsson E. (2010). miR-148 regulates Mitf in melanoma cells. PLoS ONE.

[65] Ghafouri-Fard S., Gholipour M., Taheri M. (2021). MicroRNA Signature in Melanoma: Biomarkers and Therapeutic Targets. Front. Oncol..

[66] Kawakami A., Fisher D.E. (2017). The master role of microphthalmia-associated transcription factor in melanocyte and melanoma biology. Lab. Investig..

[67] Ho Y.J., Anaparthy N., Molik D., Mathew G., Aicher T., Patel A., Hicks J., Hammell M.G. (2018). Single-cell RNA-seq analysis identifies markers of resistance to targeted BRAF inhibitors in melanoma cell populations. Genome Res..

[68] Estrela J.M., Salvador R., Marchio P., Valles S.L., López-Blanch R., Rivera P., Benlloch M., Alcácer J., Pérez C.L., Pellicer J.A. (2019). Glucocorticoid receptor antagonism overcomes resistance to BRAF inhibition in BRAF(V600E)-mutated metastatic melanoma. Am. J. Cancer Res..

[69] Lee E.F., Harris T.J., Tran S., Evangelista M., Arulananda S., John T., Ramnac C., Hobbs C., Zhu H., Gunasingh G. (2019). BCL-XL and MCL-1 are the key BCL-2 family proteins in melanoma cell survival. Cell Death Dis..

[70] Trisciuoglio D., Del Bufalo D. (2021). New insights into the roles of antiapoptotic members of the Bcl-2 family in melanoma progression and therapy. Drug Discov. Today.

[71] Liu D., Wang S., Bindeman W. (2017). Clinical applications of PD-L1 bioassays for cancer immunotherapy. J. Hematol. Oncol..

[72] Long G., Larkin J., Ascierto P., Hodi F., Rutkowski P., Sileni V., Hassel J., Lebbe C., Pavlick A., Wagstaff J. (2016). PD-L1 expression as a biomarker for nivolumab (NIVO) plus ipilimumab (IPI) and NIVO alone in advanced melanoma (MEL): A pooled analysis. Ann. Oncol..

[73] Madore J., Vilain R.E., Menzies A.M., Kakavand H., Wilmott J.S., Hyman J., Yearley J.H., Kefford R.F., Thompson J.F., Long G.V. (2015). PD-L1 expression in melanoma shows marked heterogeneity within and between patients: Implications for anti-PD-1/PD-L1 clinical trials. Pigment. Cell Melanoma Res..

[74] Carlino M.S., Long G.V., Schadendorf D., Robert C., Ribas A., Richtig E., Nyakas M., Caglevic C., Tarhini A., Blank C. (2018). Outcomes by line of therapy and programmed death ligand 1 expression in patients with advanced melanoma treated with pembrolizumab or ipilimumab in KEYNOTE-006: A randomised clinical trial. Eur. J. Cancer.

[75] Larkin J., Chiarion-Sileni V., Gonzalez R., Grob J.J., Rutkowski P., Lao C.D., Cowey C.L., Schadendorf D., Wagstaff J., Dummer R. (2019). Five-Year Survival with Combined Nivolumab and Ipilimumab in Advanced Melanoma. N. Engl. J. Med..

[76] Wolchok J.D., Chiarion-Sileni V., Gonzalez R., Rutkowski P., Grob J.J., Cowey C.L., Lao C.D., Wagstaff J., Schadendorf D., Ferrucci P.F. (2017). Overall Survival with Combined Nivolumab and Ipilimumab in Advanced Melanoma. N. Engl. J. Med..

[77] Shin D.S., Zaretsky J.M., Escuin-Ordinas H., Garcia-Diaz A., Hu-Lieskovan S., Kalbasi A., Grasso C.S., Hugo W., Sandoval S., Torrejon D.Y. (2017). Primary Resistance to PD-1 Blockade Mediated by JAK1/2 Mutations. Cancer Discov..

[78] Zhou J., Gong Z., Jia Q., Wu Y., Yang Z.-Z., Zhu B. (2018). Programmed death ligand 1 expression and CD8+ tumor-infiltrating lymphocyte density differences between paired primary and brain metastatic lesions in non-small cell lung cancer. Biochem. Biophys. Res. Commun..

[79] Lee J.H., Shklovskaya E., Lim S.Y., Carlino M.S., Menzies A.M., Stewart A., Pedersen B., Irvine M., Alavi S., Yang J.Y.H. (2020). Transcriptional downregulation of MHC class I and melanoma de-differentiation in resistance to PD-1 inhibition. Nat. Commun..

[80] Russo M., Siravegna G., Blaszkowsky L.S., Corti G., Crisafulli G., Ahronian L.G., Mussolin B., Kwak E.L., Buscarino M., Lazzari L. (2016). Tumor Heterogeneity and Lesion-Specific Response to Targeted Therapy in Colorectal Cancer. Cancer Discov..

[81] Hauschild A., Grob J.-J., Demidov L.V., Jouary T., Gutzmer R., Millward M., Rutkowski P., Blank C.U., Miller W.H., Kaempgen E. (2012). Dabrafenib in BRAF-mutated metastatic melanoma: A multicentre, open-label, phase 3 randomised controlled trial. Lancet.

[82] Chapman P.B., Hauschild A., Robert C., Larkin J.M., Haanen J.B., Ribas A., Hogg D., Hamid O., Ascierto P.A., Testori A. (2012). Updated overall survival (OS) results for BRIM-3, a phase III randomized, open-label, multicenter trial comparing BRAF inhibitor vemurafenib (vem) with dacarbazine (DTIC) in previously untreated patients with BRAFV600E-mutated melanoma. Am. Soc. Clin. Oncol..

[83] Larkin J., Ascierto P.A., Dréno B., Atkinson V., Liszkay G., Maio M., Mandalà M., Demidov L., Stroyakovskiy D., Thomas L. (2014). Combined vemurafenib and cobimetinib in BRAF-mutated melanoma. N. Engl. J. Med..

[84] Long G.V., Stroyakovskiy D., Gogas H., Levchenko E., De Braud F., Larkin J., Garbe C., Jouary T., Hauschild A., Grob J.-J. (2015). Dabrafenib and trametinib versus dabrafenib and placebo for Val600 BRAF-mutant melanoma: A multicentre, double-blind, phase 3 randomised controlled trial. Lancet.

[85] Johnson D.B., Menzies A.M., Zimmer L., Eroglu Z., Ye F., Zhao S., Rizos H., Sucker A., Scolyer R.A., Gutzmer R. (2015). Acquired BRAF inhibitor resistance: A multicenter meta-analysis of the spectrum and frequencies, clinical behaviour, and phenotypic associations of resistance mechanisms. Eur. J. Cancer.

[86] Salama A.K., Flaherty K.T. (2013). BRAF in Melanoma: Current Strategies and Future DirectionsBRAF in Melanoma. Clin. Cancer Res..

[87] Salama A.K., Flaherty K.T. (2018). Nivolumab plus ipilimumab or nivolumab alone versus ipilimumab alone in advanced melanoma (CheckMate 067): 4-year outcomes of a multicentre, randomised, phase 3 trial. Lancet Oncol..

[88] Gandhi L., Rodríguez-Abreu D., Gadgeel S., Esteban E., Felip E., De Angelis F., Domine M., Clingan P., Hochmair M.J., Powell S.F. (2018). Pembrolizumab plus Chemotherapy in Metastatic Non–Small-Cell Lung Cancer. N. Engl. J. Med..

[89] Motzer R.J., Tannir N.M., McDermott D.F., Frontera O.A., Melichar B., Choueiri T.K., Plimack E.R., Barthélémy P., Porta C., George S. (2018). Nivolumab plus ipilimumab versus sunitinib in advanced renal-cell carcinoma. N. Engl. J. Med..

[90] Lee J.B., Ha S.-J., Kim H.R. (2021). Clinical Insights Into Novel Immune Checkpoint Inhibitors. Front. Pharmacol..

[91] Carvajal R.D., Antonescu C.R., Wolchok J.D., Chapman P.B., Roman R.-A., Teitcher J., Panageas K.S., Busam K.J., Chmielowski B., Lutzky J. (2011). KIT as a therapeutic target in metastatic melanoma. JAMA.

[92] Haas L., Elewaut A., Gerard C.L., Umkehrer C., Leiendecker L., Pedersen M., Krecioch I., Hoffmann D., Novatchkova M., Kuttke M. (2021). Acquired resistance to anti-MAPK targeted therapy confers an immune-evasive tumor microenvironment and cross-resistance to immunotherapy in melanoma. Nat. Cancer.

[93] Jiang X., Zhou J., Giobbie-Hurder A., Wargo J., Hodi F.S. (2013). The Activation of MAPK in Melanoma Cells Resistant to BRAF Inhibition Promotes PD-L1 Expression That Is Reversible by MEK and PI3K Inhibition. Clin. Cancer Res..

[94] Vidotto T., Saggioro F.P., Jamaspishvili T., Chesca D.L., Picanço de Albuquerque C.G., Reis R.B., Graham C.H., Berman D.M., Siemens D.R., Squire J.A. (2019). PTEN-deficient prostate cancer is associated with an immunosuppressive tumor microenvironment mediated by increased expression of IDO1 and infiltrating FoxP3+ T regulatory cells. Prostate.

[95] Atkins M.B., Larkin J. (2016). Immunotherapy Combined or Sequenced With Targeted Therapy in the Treatment of Solid Tumors: Current Perspectives. JNCI J. Natl. Cancer Inst..

[96] Ascierto P.A., Margolin K. (2014). Ipilimumab before BRAF inhibitor treatment may be more beneficial than vice versa for the majority of patients with advanced melanoma. Cancer.

[97] Lovly C.M., Salama A.K.S., Salgia R. (2016). Tumor Heterogeneity and Therapeutic Resistance. Am. Soc. Clin. Oncol. Educ. Book.

[98] Reijers I.L.M., Menzies A.M., van Akkooi A.C.J., Versluis J.M., van den Heuvel N.M.J., Saw R.P.M., Pennington T.E., Kapiteijn E., van der Veldt A.A.M., Suijkerbuijk K.P.M. (2022). Personalized response-directed surgery and adjuvant therapy after neoadjuvant ipilimumab and nivolumab in high-risk stage III melanoma: The PRADO trial. Nat. Med..

[99] Gracie L., Pan Y., Atenafu E.G., Ward D.G., Teng M., Pallan L., Stevens N.M., Khoja L. (2021). Circulating tumour DNA (ctDNA) in metastatic melanoma, a systematic review and meta-analysis. Eur. J. Cancer.

[100] Rozenblatt-Rosen O., Regev A., Oberdoerffer P., Nawy T., Hupalowska A., Rood J.E., Ashenberg O., Cerami E., Coffey R.J., Demir E. (2020). The Human Tumor Atlas Network: Charting Tumor Transitions across Space and Time at Single-Cell Resolution. Cell.

[101] Snyder M.P., Lin S., Posgai A., Atkinson M., Regev A., Rood J., Rozenblatt-Rosen O., Gaffney L., Hupalowska A., Satija R. (2019). The human body at cellular resolution: The NIH Human Biomolecular Atlas Program. Nature.

